# Notes of Professor Gaston’s Lecture on Malignant Tumors

**Published:** 1887-06

**Authors:** J. E. Kintz

**Affiliations:** Somerset, Ohio


### Southern Medical Record.

A MONTHLY JOURNAL OF PRACTICAL MEDICINE.

☞All Communications must be addressed to R. C. WORD, M. D., Managing Edito

"*Quicquid Præcipies Esto Brevis.*"

VOL. XVII. ATLANTA, GA., JUNE, 1887. No. 6.

### ORIGINAL AND SELECTED ARTICLES.

### NOTES OF PROFESSOR GASTON'S LECTURE ON MALIGNANT TUMORS.

BY J. E. KINTZ, M. D., OF SOMERSET, OHIO.

[Concluded from our last number.]

EPITHELIOMA.

This class of affections was formerly known under the name of scirrhus, from the supposed identity with that disease. They comprise the various forms of malignant disease of the cutaneous and mucous tissues, particularly carcimona of the lip, gums, tongue, face, anus, and penis. They are not, however, limited to these parts, but sometimes invade deeper structures, as bones, muscles, lymphatic ganglions, liver, and lungs. The names by which these formations are generally known are Epithelioma cancroid and Epithelial cancer. Epithelioma is more common in men than in women, the latter being more liable to scirrhus. In the male, the lip and penis are most frequently attacked. What is called Chimney-sweep's cancer is a form of epithelioma of the scrotum. In whatever form it may show itself it does not often make its appearance before the age of thirty-five or forty. It usually occurs in one part of the body, only its origin being much less connected with the constitution than the other varieties of carcinoma. The causes of this disease are not very well understood. It is sometimes directly traceable to an external injury, as long-continued pressure. Epithelioma of the lip is often charged to smoking. The disease sometimes manifests a hereditary tendency. Cancroid generally begins in the form of a tubercle, crack or wart-like excrescence, hard to the touch, movable, and somewhat tender on pressure. When ulceration sets in the exposed surface has a foul, unhealthy, fungated appearance, with irregular granulated edges and a hard, rough base. The discharge is generally abundant and of a thin, sanious, acrid description, often eroding the skin in the neighborhood of the part affected. The pain in this disease is of a sharp, darting or pricking nature, often extending to the surrounding parts. During the progress of the disease lymphatic involvement occurs, sometimes early, but generally not until rather late in the disease.

In making section of epithelioma it is found to grate under the knife. Minutely examined, it is found to consist of cells not unlike pavement epithelium, only they are larger. The cells vary considerably in shape ; they usually have one or two small nuclei. Mixed up with the cells of epithelioma are great numbers of free nuclei and granules, and sometimes crystals of cholesterine pigment cells, and blood corpuscles. It seems that this disease is deficient in some of the elements of ordinary cancer. Epithelial cancer is generally tardy in its growth, cancer of the lip often lasting a number of years before it terminates fatally.

### COLLOID.

The name of this class of morbid growth has reference to the jelly-like appearance of one of its principal constituents. It has sometimes been described as gelatiniform, alveolar, and gum cancer. The favorite seat of this morbid growth are the omentum, stomach, rectum, ovary, and lower jaw, subcutaneous cellular tissue, and the bones of the extremities. The testicle, mamma, uterus and lymphatic ganglions are sometimes attacked. It is never found in the eye and its appendages. It takes place in both sexes and at all periods of life, after puberty, but is most common between the ages of thirty-five and fifty. Colloid usually occurs under two forms, as tumor and as an infiltration, the latter most common in the alimentary canal, particularly the stomach and rectum ; the former in glandular organs, peritoneum and omentum, ovary cellular tissues and bones.

When colloid occurs as a distinct tumor it is of a globular, rounded or irregular shape, and of firm consistence. It varies in size from that of a marble to that of an adult head. The knife, in passing through it, makes a creaking sound. Colloid consists of two elements—a stroma and a peculiar jelly-like matter, from which it takes its name; the stroma consists of fibres, and are arranged in such a manner as to form cells or cavities from the size of a pin-head to that of a small marble. The stroma seems to be a new formation, and possesses great firmness and density; it is of a whitish, grayish or pale yellowish color. The fibrous matter of colloid is almost destitute of free cellular substance; it receives an abundant supply of vessels. The manner in which the vascular system is arranged is not well determined. The other element is an unorganized product of a whitish, greenish, or yellowish color and of the consistance of ordinary jelly; sometimes it is as firm as moist cheese or the white of hard-boiled eggs; then, again, it sometimes resembles currant jelly or a solution of gum shellac. Cancer cells, such as are found in scirrhus and encephaloid, are occasionally found in colloid, but not as a necessary constituent. Colloid seems to have less disposition to ulcerate, and contaminate the surrounding lymphatic ganglions than the other heterologous formations.

### MELANOSIS.

Melanosis, sometimes called Black Cancer, occurs most commonly in the cellulo-adipose tissue beneath the skin, in the folds of the mesentery and omentum, in the mediastinal cavities, the lymphatic ganglions, the eye, liver, lungs, and parotid glands; it is also met with, sometimes, in the serous and fibrous membrane, bones, ovaries, heart, pancreas, and spleen. It sometimes co-exists with some of the other heterologous deposits. It takes place in both sexes and at all periods of life, but is most frequent in adult and middle-aged subjects. Melanotic matter is deposited in several varieties of form, the tuberoid being the most common. It occurs in small masses of a rounded, ovoidal or irregular shape, with or without a cyst, from the size of a pin-head to that of a walnut. By the union of these small masses large tumors are sometimes found, reaching the size of a fist or even a fœtal head. Melanotic matter, in a pure state is of a sooty-black or dark-brown color; in consistence it varies from that of ink to that of fibro-cartilage. Its chemical constituents are albumen-fibrin and a dark substance not unlike the coagula of blood, with a small quantity of iron, soda, magnesia, lime, and potash; it imparts a characteristic stain to linen, and resists decomposition. When examined microscopically, melanotic matter is found to consist of a fibrous net-work inclosing numerous meshes filled with free pigment cells of a pale-yellowish, dark or dark-brown color, and of a rounded, oval or irregular shape. The nature and cause of this disease is not well understood. When disintegration takes place there is found a foul, non-granulating, unhealthy ulcer; the discharge is of a sanious character, mixed with heteroclite secretions. It is generally of slower growth than that of scirrhus or encephaloid ulcers, when it is found in a number of organs at the same time. The general health often becomes impaired before ulceration takes place, the patient assuming a sallow, haggard look.

### DIAGNOSIS.

Epithelial cancer, usually, is not difficult to diagnose. Its situation at the junction of the skin and mucous membrane, or upon either of these structures, its origin in a crack or fissure, or wart-like excressence; its great firmness, its slow growth, and small size, and the absence for a long time of pain or any constitutional taint. The principal sign by which we may know melanosis is the peculiarity of its color. Colloid tumors may be confounded with fibrous and enchondromatous formations.

A careful examination of the morbid mass will be likely to assist a correct diagnosis.

About the only disease with which encephaloid could be confounded is scirrhus, but there are several points of difference which, when considered, will greatly aid the diagnosis:

1st. Encephaloid is soft and elastic to the touch, not uniformly but more so at some points than at others. Scirrhus is uniformly hard and inelastic.

2d. Encephaloid grows rapidly and soon acquires a large bulk; scirrhus grows more slowly, and the bulk is comparatively small.

3d. In encephaloid the pain is slight till ulceration begins; in scirrhus the pain begins early, and is distinctly localized, and is of a sharp, darting character.

4th. In encephaloid there is always marked enlargement of the veins; in scirrhus the vessels retain their natural size, usually.

5th. In encephaloid the ulcer is foul and fungous, with thin, undermined and livid edges, and it is subject to frequent hemorrhages; in scirrhus the ulcer is incrusted with spoiled lymph, has steep, abrupt edges, bleeding very little and not often.

6th. In encephaloid there is generally early involvement of lymphatics; in scirrhus, generally not until late.

7th. Encephaloid occurs at all periods of life; scirrhus seldom before the age of forty or forty-five.

8th. Encephaloid is most frequent in the eye, testicle, mamma, lymphatic ganglion, bones and cellular tissue; scirrhus is never found in the eye and testicle, and seldom in lymphatics and bone.

9th. Encephaloid usually terminates fatally in from nine to twelve months ; scirrhus seldom sooner than eighteen months or two years.

The microscope is a great aid in forming a diagnosis of malignant tumors, but it is not always an infallible one.

### TREATMENT.

The surgeon should direct his attention to the patient's diet, bowels, and secretions, and to the avoidance of all sources of local irritation. The diet should be of a bland, unirritating character, but sufficiently nutritious to preserve a sound condition of the blood and to maintain the tone of the muscular system. A milk diet seems to answer better than any other. Bowels should be kept in a soluble condition, but all active purgation should be avoided. Sleep should be procured, and pain allayed, by anodynes. If debility exist, tonics are to be used, such as iron, quinine, etc. Night sweats may be controlled by aromatic sulphuric acid. Care should be taken to keep the affected part at rest, and free from pressure and excitement. When the part takes on an ulcerative action the resulting one should be kept clean by frequent ablutions.

The treatment of cancerous disease by compression was for a long time considered beneficial, but of late years it has fallen into disuse. The great indication is for excision when a growth is suspected to be malignant, not that we may expect a permanent cure but it often gives a temporary relief for a number of months or even years. There are cases on record in which the removal of epithelioma has seemed to result in a cure.

There are some general rules for the excision of malignant growths that it may be well to observe :

1. It is necessary that the incisions should be carried through the healthy tissues at some distance from the morbid deposit, and care taken that every part of the diseased structure be removed.

2. The surgeon should endeavor to preserve as much as possible of the common integument, in order to afford a complete covering to the surface of the wound.

3. When only a portion of an organ is involved by the heteromorphous matter the rule is to remove, not a part but the whole of it.

4. In removing malignant tumors the surgeon should alwsys endeavor to avoid the loss of blood.

5. The whole of the wound left by the operation should be healed by first intention, and dressing should be to that end.

6. When the integument is deficient it is practicable sometimes to know the requisite amount from the surrounding parts.

7. When a sufficiency of integument cannot be obtained it is sometimes recommended to cauterize the whole raw surface, so as to form a superficial eschar.

The treatment, after the operation, should be upon general principles. Everything should be done to insure union by first intention. When the patient has recovered from the immediate effects of the operation. he should be put on treatment intended to maintain his health as near the normal standard as possible. Above all, strict attention should be paid to his diet. A milk diet seems to fill the indication better than any other.

There are some circumstances under which surgical interference is contra-indicated :

1. No operation should be performed when the disease is congenital, or manifests itself soon after birth.

2. Interference should be avoided when the disease exists in several parts of the body.

3. Operation is never resorted to when the morbid growth has attained an unusual size ; when there is serious local involvement, or when there is marked evidence of cancerous cachexia.

4. When the disease advances very rapidly and breaks through its original boundary into the surrounding tissues, as a general rule an operation will not be attended with very satisfactory results.

5. A quickened pulse, occasioned by local irritation, usually indicates an unfavorable result if an operation be performed.

6. Latent cancer should not be tampered with.

7. An operation should not be performed when there is serious disease of an important internal organ.

Great relief is sometimes found to result from the use of arsenical preparations. It will sometimes change the character of the disease and accomplish good results. Commence with small doses and gradually increase until a grain and a half is reached, daily.

